# Supplementary material for: Prediction of African Swine Fever Virus Inhibitors by Molecular Docking-Driven Machine Learning Models
Source: Molecules. 2021 Jun 11;26(12):3592. doi: 10.3390/molecules26123592 (PMC8231271; doi:10.3390/molecules26123592)
Supplement: Supplementary file 1 [file molecules-26-03592-s001.zip › molecules-1206933-supplementary.pdf]

Prediction of African swine fever virus inhibitors by molecular docking-driven machine learning models

Jiwon Choi[1, 2*], Jun Seop Yun[1], Hyeeun Song[1], Yong-Keol Shin[3], Young-Hoon Kang[6], Palinda Ruvan Munashingha[3], Jeongyeon Yoon[3], Nam Hee Kim[1], Hyun Sil Kim[1], Jong In Yook[1, 2], Dongseob Tark[4], Yun-Sook Lim[5*], and Soon B. Hwang[5,6]

[1]Department of Oral Pathology, Oral Cancer Research Institute, Yonsei University College of Dentistry, Seoul, Korea, [2] Met Life Sciences Co., Ltd., Seoul, Korea, [3]Enzynomics, Yuseong-gu, Daejeon 34050, Korea, [4]Laboratory for Infectious Disease Prevention, Korea Zoonosis Research Institute, Jeonbuk National University, Iksan, Korea, [5]Laboratory of RNA Viral Diseases, Korea Zoonosis Research Institute, Jeonbuk National University, Iksan Korea, [6]Ilsong Institute of Life Science, Hallym University, Seoul, Korea

[*] Corresponding authors.

Jiwon Choi, *E-mail*: edccjw@gmail.com

Yun- Sook Lim, *E-mail*: yunsolim@hanmail.net

Jiwon Choi and Jun Seop Yun contributed equally to this work

Supplementary Materials

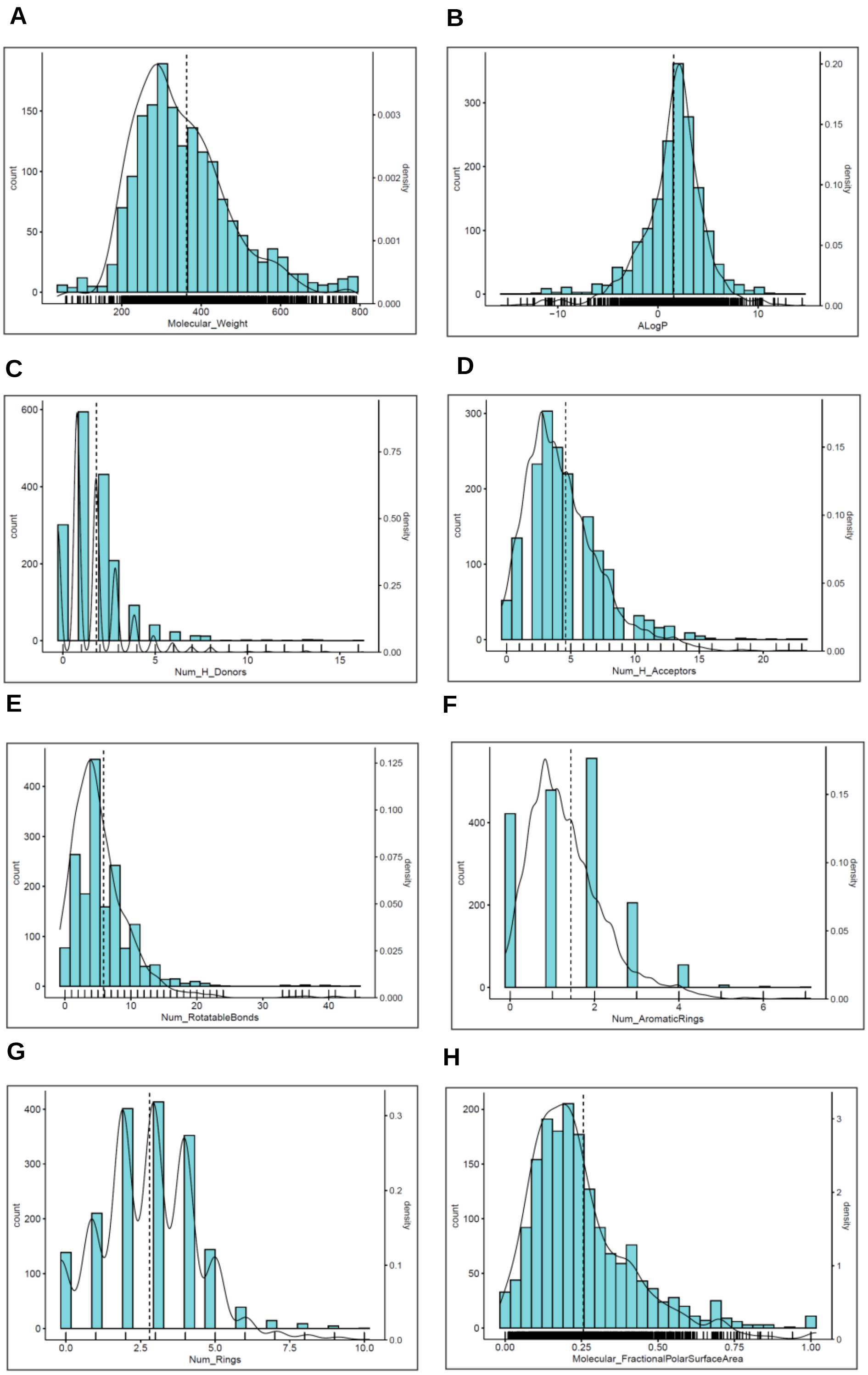

Figure S1: Chemical properties of the compounds from the dataset are compared using histogram for eight molecular descriptors. The dotted lines represent mean values and all histograms were generated with the R software.

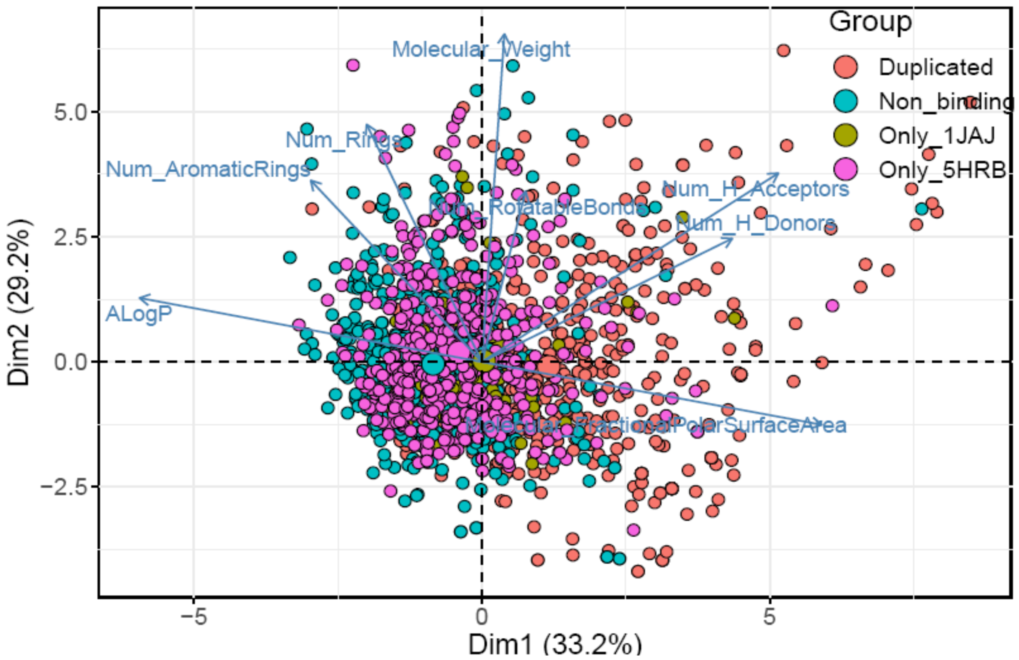

Figure S2: The chemical space distributions based on the principal component analysis. Data points are color-coded by each dataset. The loading plot vectors are represented by arrows for each physicochemical property

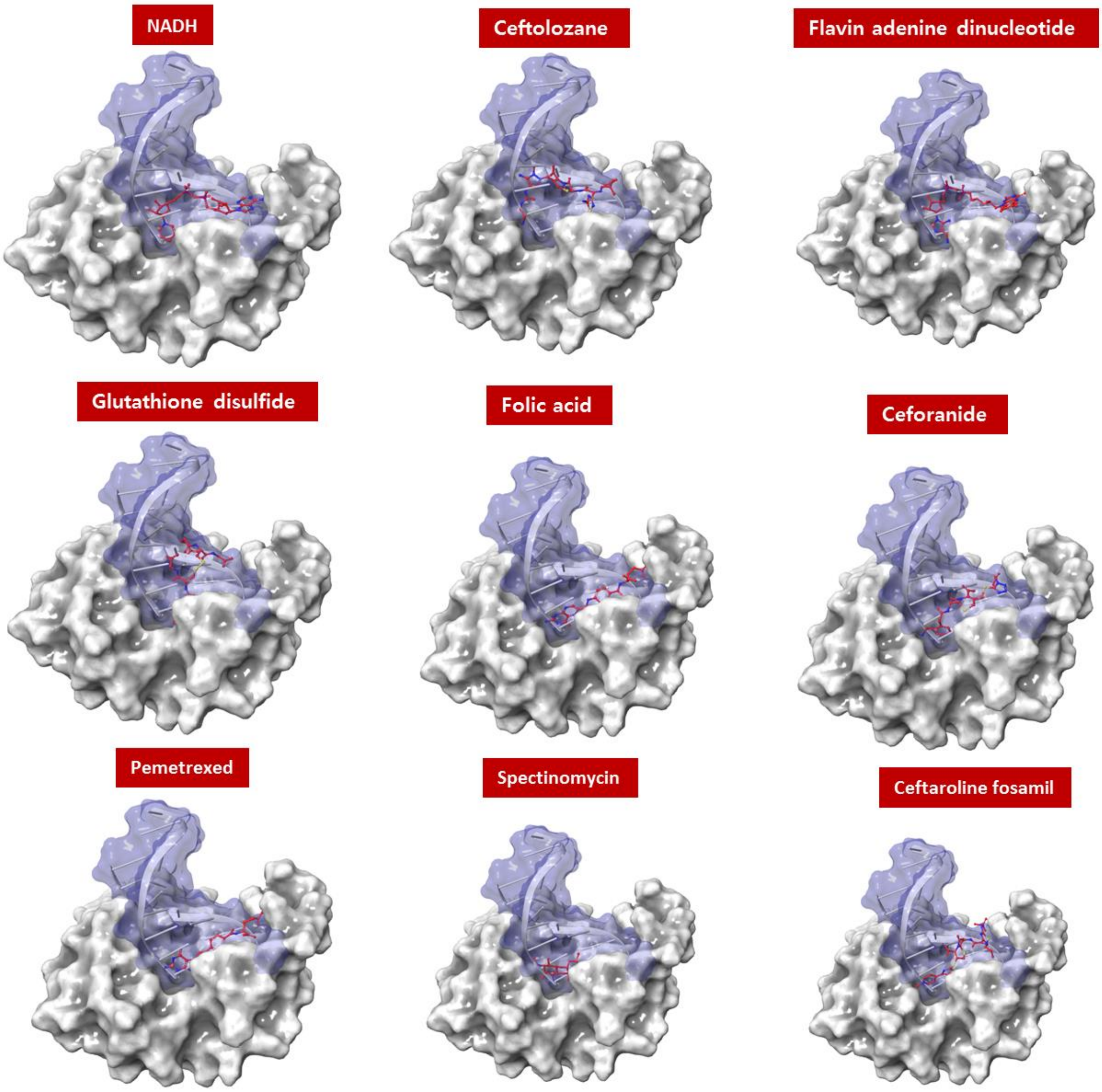

Figure S3: The predicted binding pose of top ranked nine ligands to AsfvPolX protein

Table S1. The list of top-ranked ten ligands in Cluster 2

No	DrugBank_ID	Name	Dockscore (5HRB)	Dockscore (1JAJ)
1	DB00157	NADH	-7.995	-5.774
2	DB09050	Ceftolozane	-7.769	-6.578
3	DB03147	Flavin adenine dinucleotide	-7.688	-5.978
4	DB03310	Glutathione disulfide	-7.638	-6.784
5	DB00183	Pentagastrin	-7.495	-6.746
6	DB00158	Folic acid	-7.428	-6.161
7	DB00923	Ceforanide	-7.364	-6.53

8	DB00642	Pemetrexed	-7.25	-5.854
9	DB00919	Spectinomycin	-7.243	-4.429
10	DB06590	Ceftaroline fosamil	-7.016	-5.421
